# Approach to childhood interstitial lung disease in resource limited setting

**DOI:** 10.11604/pamj.2018.31.153.15568

**Published:** 2018-10-31

**Authors:** Varsha Vekaria-Hirani, Adil Waris

**Affiliations:** 1 Paediatric Department, MP Shah Hospital, Nairobi, Kenya; 2 Paediatric Department, Agakhan University Hospital, Nairobi, Kenya

**Keywords:** ChILD, resource limited setting, Sub-Saharan Africa, children

## Abstract

Childhood interstitial lung disease (ChILD) is a rare disease and sensitization is needed in the recognition, diagnosis and treatment approaches. There is no formal approach to diagnosis or therapy in resource limited regions. We present a case of a 4-month infant who presented with all the criteria for diagnosis of ChILD. Lung biopsy being the gold standard is a challenge in our setting and diagnosis was based on clinical signs and imaging after ruling out of other similar respiratory conditions by way of individual trials of therapy. Monotherapy with prednisone showed clinical improvement within days of initiation.

## Introduction

Childhood interstitial lung disease (ChILD) is a rare group of disorders characterized by diffuse lung infiltrates and impaired gas exchange. There has been progress in the approach to ChILD, particularly in its recognition and classification in infants being discrete from older children. Many of the forms may be triggered by an inflammatory process such as an infection [1]. The epidemiology is poorly described and most often appear as case reports. One large study by Dinwiddie *et al.*, in the United Kingdom and Ireland (1995- 1998) showed estimated prevalence of 3-6 per million in immunocompetent children [2]. In recent years advancement of knowledge on ChILD has developed in to a more formal classification system [3]. The causes range from diffuse developmental problem (acinar, alveolar and capillary dysplasia), alveolar growth abnormalities, genetic surfactant dysfunction disorders, pulmonary interstitial glycogenesis, neuroendocrine cell hyperplasia of infancy and others. However, these existing classifications may not be useful in the resource limited setting where lung biopsy and genetic testing for inherited surfactant disorders is not readily available. Environmental factors may play a role altering the disease hence differing from the temperate regions. A high index of suspicion is required in diagnosis. The diagnosis is based largely on history, examination, imaging (chest X-ray (CXR) and high-resolution chest computed tomography (CT) scan), bronchoalveolar lavage assessment, genetic testing and lung biopsy [3]. An infant who presents with largely afebrile chronic cough with tachypnoea and fine crackles on auscultation, hypoxemia and diffuse abnormality on imaging for more than a month and not responding to standard therapy for chronic respiratory disease for example atypical pneumonia, pneumocystis jiroveci pneumonia, gastroesophageal reflux, aspiration syndromes, cystic fibrosis, fungal infections, tuberculosis and HIV should be evaluated for ChILD. Few guidelines on management have been developed such as European guideline [4]. Prednisone and hydroxychloroquine are used in management. Current research on macrolide (azithromycin), methotrexate, azathioprine, cyclosporine and rituximab are ongoing.

## Patient and observation

We present a 4-month female patient who has been unwell from 2 weeks of life. The baby was delivered at term with weight of 3.0 kg and discharged uneventfully. At 2 weeks of age developed cough that progressively worsened over a week with post tussive vomiting and no fevers. The baby was able to breastfeed, had no breathing difficulties or worsening of cough during feeds. There was no contact with persons with chronic cough or pulmonary tuberculosis (TB). There was no family history of smoking and biomass fuel use. She was treated thrice for a probable bacterial infection with amoxicillin-clavulanic acid, erythromycin and trimethoprim-sulfamethoxazole respectively with no benefit as an outpatient. The infant worsened and was admitted at 1-month age. Clinically was wasted (weight 2.2kg; weight to height z score of -3SD), afebrile, had tachycardia and was in severe respiratory distress (respiratory rate (RR)= 78/minute, use of accessory muscles and oxygen saturation (SaO2) of 40% off oxygen). On auscultation had bilateral equal air entry with crackles. The other systems were normal. An initial impression of possible aspiration pneumonia was made. Septic screen, TB screen, HIV, Cytomegalo virus (CMV) antibodies and fecal elastase were negative. Vitamin D3 level, echocardiogram and barium swallow were normal. The initial chest radiographs were reported as bronchopneumonia and with subsequent radiographs showing ground glass appearance, a thought of ChILD was considered. With no improvement since a week of admission, CT scan of the chest was done that showed ground glass appearance, air bronchograms and pleural thickening in the posterior recesses and no lymphadenopathy. The infant initially was started on nebulization (3% hypertonic saline and levo-salbutamol), esomeprazole, domperidone and supportive care. With no improvement various antibiotics and antifungal were given over the following month (cefuroxime, vancomycin, meropenem, amikacin and clarithromycin and fluconazole). At the age of 3 months was started on 1.5mg/kg/24 hours of prednisone and other medications were stopped. Clinical improvement was noted within a week of treatment; the RR was 55/minute with normal breath sounds and no crackles. The SaO_2_ was 95-99% on nasal prongs at 2L/min, had weight gain and was able to breast feed. The dose was tapered down to 1mg/kg/24 hours and a week later gained weight to 3 kg, RR= 45/minute and stay off oxygen for 2 hours with SaO_2_ > 95%. While the patient was recuperating in hospital, developed probable nosocomial pneumonia 26 days after starting prednisone and died.

## Discussion

**Figure 1:**
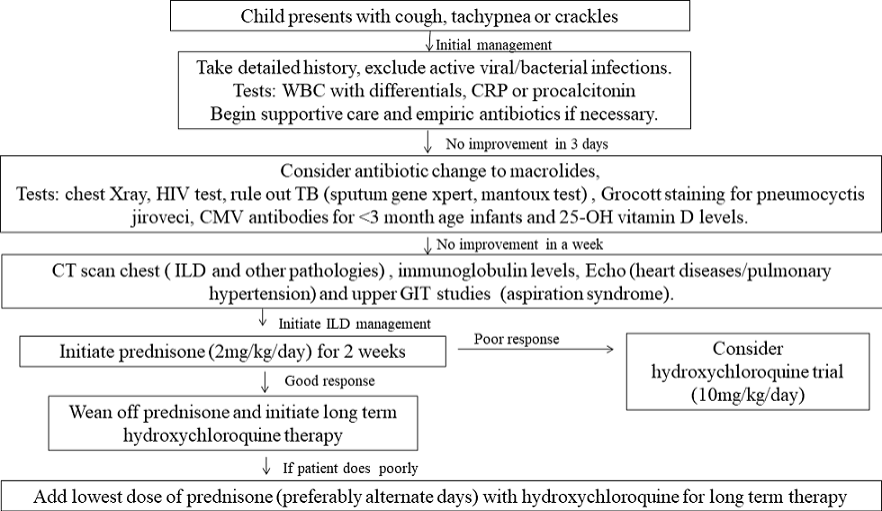
Approach to ChILD in a resource limited setting

Interstitial lung disease in children is a rare disease and the diagnosis is usually delayed such as in our case where the infant was diagnosed with ChILD at 3 months of age. ChILD occurs in the developing world but is under reported and confused with resistant bacterial or viral pneumonia. The diagnosis in our case was made after ruling out all possible causes of the respiratory distress. As we note, the infant was managed initially for an infection (bacteria, viral bronchiolitis, pneumocystis jiroveci and fungal) and gastroesophageal reflux with no improvement. Cystic fibrosis is rare in our set up and was ruled out using fecal elastase level as sweat test is unavailable in our setting. There are few specialized cardiothoracic surgeons trained to do lung biopsy but due to bed space availability and required resources for post biopsy care and monitoring, lung biopsy is a challenge in our setting. Furthermore, there is lack of expertise in staining and histological evaluation of lung tissue or bronchoalveolar specimen to identify the pathology causing ChILD. Genetic tests to determine surfactant mutations in proteins (SP-B, SP-C, and ABCA3) are also not available in sub Saharan Africa. ChILD cannot be evaluated by the recommended protocols in the developing African region due to unavailability of expertise, equipment and finances, hence the approach described in Figure 1 has been suggested by the authors. A high suspicion is required in diagnosis of ChILD, with good knowledge on fetal lung development, pulmonary pathology and genetic abnormalities. In our case, our infant had all signs and radiological evidence of ChILD, hence sensitization to this condition would reduce morbidity and mortality. In the sub-Saharan countries, most lung pathology is from infectious pathology and hence the diagnosis was delayed. Radiological imaging has shown to greatly assist in diagnosis of ChILD. Persistence of linear reticular or nodular pattern while on treatment for infectious or reflux should raise a suspicion of ChILD. In our case progressive worsening of ground glass appearance was present on both CXR and CT scan of the lungs. In such a scenario ChILD should be ruled out as described by Burtsiva *et al.* [5]. Our infant in this case showed improvement within a week of starting prednisone. There are no studies on empiric treatment choice in the sub-Saharan region, which is a subject that needs to be explored. Currently we begin with a steroid based protocol as part of trial of therapy with eventual move to hydroxychloroquine. In the developed countries, there is more focus where support programs have been developed such as ChILD Foundation which needs to have similar replication in resource limited settings. We face prolonged and unnecessary hospitalization with huge consumption of limited resources and associated unnecessary antibiotic abuse as in our case where the child has been in hospital for over 2 months. Family support is critical as shown in a study done by Carlee *et al.* as these children have difficulty in feeding and they require a lot of psychological support [6].

## Conclusion

There are no diagnostic and management protocols in the sub-Saharan set up and it is hoped that this article can be a start. There is need for more sensitization on ChILD in sub-Saharan Africa. It being a rare disease may go unreported with delays in diagnosis and associated high mortality. More research is needed to classify the causes of ChILD in the sub-Saharan region as the causes may be different from those in temperate regions.

## Competing interests

The authors declare no conflict of interests.
